# Acute uterine inversion – A complication revisited; a case series and review of literature

**DOI:** 10.1515/crpm-2020-0081

**Published:** 2022-06-27

**Authors:** Amanjot Kaur, Beant Singh

**Affiliations:** Department of Obstetrics and Gynaecology, Government Medical College, Patiala, Punjab, India

**Keywords:** acute uterine inversion, neurogenic shock, postpartum hemorrhage, uterine inversion

## Abstract

**Objectives:**

The objective of this case series is to discuss the various presentations of acute uterine inversion and to discuss how these varied presentations can cause a diagnostic confusion. Differences in acute uterine inversion following a vaginal delivery and a cesarean section are also discussed along with the management of acute uterine inversion, emphasizing the need for a rapid diagnosis and management.

**Case presentation:**

Three such cases of acute uterine inversion – two after vaginal delivery (one second-degree inversion and one third degree inversion) and one during cesarean section have been discussed along with their management.

**Conclusions:**

Uterine inversion is a potentially life-threatening complication which can be prevented by active and careful management of third stage of labor and avoiding cord traction prior to development of the signs of placental separation. Early stages of uterine inversion may be confused with a prolapsed fibroid or a cervical polyp. Prompt management can avert maternal mortality and morbidity.

## Introduction

Uterine inversion, which involves the collapse of the fundus of the uterus into the uterine cavity, is an extremely rare life-threatening obstetric complication with an incidence of one in 2,000–8,000 deliveries [1]. It usually occurs as a result of mismanaged third stage of labor and involves premature cord traction prior to placental separation by relatively untrained or unaware accoucheur. If not promptly managed, this complication may result in a very high mortality rate. We present three such cases encountered by us during our practice along with their management.

## Case presentation

### Case 1

A 20 year old P1L1 (previous one delivery with one live issue) presented to our hospital at 5 h postpartum in shock. Patient had a vaginal delivery with right mediolateral episiotomy at a peripheral hospital which was apparently uneventful. One hour postpartum, patient complained of pain in lower abdomen. On examination, patient was found to have excessive bleeding per vaginum which seemed to be uterine in origin, with tachycardia (pulse rate 110 min). The blood loss, however, was not quantified. Patient was referred after vaginal packing with three roller gauzes to our hospital. On admission, patient’s blood pressure was 90/60 mmHg and pulse rate was 130 min. The uterus was apparently well retracted with a fundal height of around 14–16 weeks with a small depression which appeared to be dimpling at the fundus. The abdomen was soft. On removal of the packs, cervix was 4 cm dilated with either the uterine fundus or a large uterine fibroid felt through the cervical ring. During the examination period, patient’s pulse rate increased to 170 min and blood pressure dropped to 70/60 mm Hg. A provisional diagnosis of uterine inversion/prolapsed fibroid with postpartum hemorrhage was made. Hemoglobin at admission was 5.4 gm% and the total leukocyte count (TLC) was 26,400 mm^3^. Keeping in mind the deteriorating vital parameters and diagnostic confusion, a decision for laparotomy was taken with the intent of doing a peripartum hysterectomy. The abdomen was opened with a midline vertical infraumblical incision and the peritoneal cavity was reached. The uterus was found to be inverted from the fundus which explained the dimpling. When evaluated vaginally, the fundus was reaching the external os of the cervix. Thus, a diagnosis of second-degree inversion was made. Combined vaginal and uterine approaches were used to reposit the uterus back to its original anatomical position. Vaginally, upward pressure was applied with a fist and abdominally, allis forceps were used for reposition. Intramuscular and intravenous oxytocin was administered for uterine retraction after reposition. Intramyometrial carboprost was later administered in view of persistent uterine atony. After the recovery of uterine tone, a bakri balloon was introduced into the uterine cavity and was inflated to a volume of 360 mL with normal saline. The procedure was done under general anesthesia with simultaneous hemodynamic resuscitation. Patient was given 1,200 mL PRBC (Packed Red Blood Cells), and noradrenaline as ionotropic support. She also received Intensive Care Unit (ICU) care for 2 days, higher antibiotics (injection piperacillin tazobactam 4.5 g i/v 8 hourly for 7 days) and one more blood transfusion in the postoperative period. She was discharged in stable condition on tenth postoperative day with hemoglobin value of 8 gm%. At the time of discharge, she was explained about the danger signs and the possible need to report back to the hospital in case of reinversion, vaginal bleeding or fever.

### Case 2

A 22 year old P1L1 (previous one delivery with one live issue) presented to us 4 h postpartum in shock. Patient had a vaginal delivery conducted at home by traditional midwife (dai). Patient had uterine inversion which may have happened due to premature cord traction during the third stage. It was diagnosed by the midwife after which she was taken to the nearest hospital. An unsuccessful attempt to reposit the uterus was made at this hospital. The vagina was packed with two roller gauzes and referred to our hospital. On admission, the blood pressure was 84/50 mm Hg, pulse rate 143 min with 99% oxygen saturation at room air. The per abdomen examination revealed a uterus around 16 weeks size with palpable depression at the fundus. Subsequently on removal of the packs, uterine fundus was felt in the vagina. The cervical ring felt well above the level of the fundus, thus confirming a diagnosis of third degree inversion. Patient was explored under general anesthesia and manual repositioning of the uterus was done. Uterotonic agents, viz, intramuscular and intravenous oxytocin and intramuscular carboprost were given, following which the uterus became well contracted. Patient received 900 mL PRBC along with intravenous fluids and noradrenaline during simultaneous resuscitation carried out by the anesthesia team. The patient was extubated 3 h post procedure. This patient also received injection piperacillin tazobactam 4.5 g i/v 8 hourly for 6 days. The post procedure period was uneventful and patient was discharged in stable condition on seventh postoperative day with the same postoperative advice as case 1.

### Case 3

A 29 year old G2P1L1 (second gravida with previous one live issue) was admitted for elective cesarean section as patient was not willing for trial of vaginal delivery after cesarean section (TOLAC) at 39 weeks gestation. After following the appropriate preoperative protocol, patient was taken up for cesarean section and baby was delivered by lower segment cesarean incision. After observing uterine contraction, gentle traction on the cord was applied to remove the placenta which resulted in a first degree inversion of the uterine fundus. It was immediately corrected but was associated with atonic postpartum hemorrhage. Intramuscular carboprost and oxytocin were given to retract the uterus. Since the inversion was immediately corrected, it was not associated with any significant deterioration of vitals. After ensuring the uterine tone, abdomen was closed in layers and a close watch on vitals was kept in the post-operative period. Patient received only the routine surgical prophylactic antibiotic (injection cefazolin 1 g) and no other higher antibiotic. She was discharged uneventfully on fourth postoperative day.

## Discussion

Uterine inversion is an acute life-threatening condition requiring urgent intervention. It is associated with high risk of mortality due to hemorrhage and shock [2]. Apart from being hemorrhagic, the shock is also believed to have a neurogenic component which is attributed to increased vagal tone from stretching of the pelvic parasympathetic nerves. These patients are at a higher risk for surgical intervention and might need hysterectomy in view of the associated atonic postpartum hemorrhage.

### Etiopathogenesis

The complication is attributed to mismanagement of the third stage of labor. The common etiological factors are premature traction on the cord or fundal pressure before placental separation. Nulliparity, grandmultiparity, magnesium sulfate use during labor, sudden increase in intrabdominal pressure (coughing, sneezing, pushing), connective tissue disorders, fundal implantation of placenta, adherent placenta, uterine structural anomalies, fetal macrosomia and short umbilical cord are other causative factors described in literature. The pathophysiology can be explained by three possible events: a portion of the uterine wall prolapses through the dilated cervix or indents forward, relaxation of part of the uterine wall, simultaneous downward traction on the fundus leading to uterine inversion [3, 4]. The etiological factors in our patients have been discussed in Table 1.

**Table 1:** Clinical details of the patients.

	Case 1	Case 2	Case 3
Parity	Primiparous	Primiparous	Multiparous
Type of delivery	Vaginal delivery	Vaginal delivery	Cesarean section
Placental attachment	Not known	Not known	Fundal
Place of delivery	Peripheral hospital	Home delivery	Tertiary care hospital
Type of inversion	Acute	Acute	Acute
Degree of inversion	Second-degree	Third degree	First degree
Atonic PPH	Yes	Yes	Yes
Development of shock	Yes	Yes	No
Need of blood transfusion	Yes	Yes	No
Volume of blood transfused	1,500 mL	900 mL	–
Technique of repositioning	Abdominal + manual repositioning	Johnsons maneuver	Manual repositioning
Need for laparotomy	Yes	No	No
Hysterectomy	No	No	No

### Classification

By virtue of timing of the complication, it can be classified as acute, subacute or chronic. Inversions diagnosed within 24 h are termed as acute, those diagnosed after 4 weeks are termed chronic and those diagnosed between 24 h and 4 weeks are termed as subacute. By virtue of degree of inversion, it can be classified from first to fourth degree inversion. All the patients discussed in this article had acute puerperal inversions. An inversion is said to be first degree when the uterine fundus inverts to the level of cervical ring but does not protrude beyond it. It crosses the cervical ring in second-degree inversion, but does not reach the perineum. The fundus reaches the perineum in third degree inversion and in fourth degree inversion, the vagina inverts along with the fundus and lies outside the introitus [2, 5]. Table 1 summarizes the clinical findings and management of the three patients.

DIAGNOSIS: The diagnosis is usually clinical. Various presentations are vaginal bleeding, lower abdominal pain, smooth, round mass protruding from the cervix or vagina, and urinary retention [6]. Majority of patients present with hemorrhage, with or without shock. Many times, the shock seems to be out of proportion to blood loss, either due to neurogenic component, or due to underestimation of the blood loss by the attending physician, or both. Maternal hemorrhage was reported to be seen in 37.7% by Coad et al. [7] and 65% by Baskett et al. [8]. However, shock was reported in 32% cases by Brar et al. [9] and 1.3% cases by Coad et al. Cases 1 and 2 developed hemorrhagic shock and required blood transfusion. Transient uterine atony was observed in the case 3, but shock was averted owing to rapid correction due to the complication occurring during a cesarean section.

Bimanual examination is the first line diagnostic tool wherein a cuplike depression is felt at the fundus associated with a congested, soft and bleeding mass in vagina. Presence of placenta attached to this mass is pathognomonic [9]. Diagnosis during cesarean section is relatively easier as the inversion occurs under direct vision. In case of first- and second-degree inversions, sometimes the diagnosis may become difficult and the clinical findings may be confused with a prolapsed fibroid or uterine polyp [3].

Imaging is used for diagnosis in circumstances where clinical examination is equivocal. On transabdominal ultrasonography, a characteristic “target sign” showing a hyperechoic fundus surrounded by a hypoechoic rim created by the space between the endometrium and vaginal walls can be seen in the transverse plane. When viewed in the sagittal plane, a y-shaped endometrium is seen in first degree inversion, and a mirror image of the uterus with the two inverted serosal surfaces giving an image of endometrial stripe can be seen. Mirroring of the uterus is seen on transvaginal sonography. 3D power doppler is a very useful modality for a quick diagnosis and works by identifying the course of uterine artery [10]. Magnetic Resonance Imaging (MRI) is used both as a diagnostic tool and for the planning of surgical procedures, especially in subacute, chronic and non-obstetric cases [11, 12]. However, there is no role of MRI in a hemodynamically unstable bleeding patient. A U-shaped uterine cavity and an inverted uterine fundus on a sagittal image and a ‘bulls-eye’ configuration on an axial image on T2-weighted scans can be observed on MRI [13].

In case 1, we had difficulty in diagnosis since it was a second-degree inversion and was confused with a prolapsed fibroid. The lack of antenatal ultrasonography records added to the confusion. Considering the inconclusive clinical findings and rapidly progressing shock, a decision for laparotomy was taken, bypassing the imaging. In case 2, classical clinical findings of fundal cupping were present and in case 3, the diagnosis was easily established by virtue of the complication occurring during a cesarean section.

### Management

Early diagnosis and prompt uterine repositioning along with aggressive resuscitation are cornerstones of management and define the prognosis in a given patient.

Both non-surgical and surgical methods for reposition of uterus have been described. Johnson described a maneuver in which surgeon’s entire hand is positioned inside the vagina and the uterus is lifted out of the pelvis and elevated above the level of the umbilicus. The whole hand along with two-thirds of the forearm is placed in the vagina. Holding the fundus in the palm and keeping the tips of the fingers at the uterocervical junction, the fundus is raised above the level of the umbilicus. The maneuver causes stretching of the uterine ligaments, relaxes and widens the cervical ring thus aiding in the repositioning [14]. Successful reduction has been described in 43–88% cases [4]. This method is easy to perform before the development of the contraction ring. After the ring develops, it is sometimes difficult to attain the desired result. Alles and Henderson modified this procedure by use of ringed forceps at the cervical ring for countertraction. It is difficult to obtain expertise in this procedure in view of the rarity of the complication.

In 1945, O’Sullivan first described the method of hydrostatic reduction. Warm normal saline is instilled into the vagina through a catheter, while the obstetrician blocks the introitus with his hand or a ventouse cup. The fluid bag is kept 100–150 cm above the vaginal level. The resulting distension of the vagina pushes the fundus upwards to its normal position by hydrostatic pressure. The pitfalls of this method include difficulty in maintaining adequate seal at the vaginal end, and possible risks of infection and embolism. The WHO recommends hydrostatic reduction for correction of inversion if manual repositioning fails. Uterine rupture should be excluded before attempting this method [15]. The success rate of this procedure has not been documented in the literature. Also, there is little guidance in literature regarding how to use the cup and how much fluid can be used for distension. The cup should not be placed over the fundus but in the direction of posterior fornix to allow distension of the vagina.

Though tocolysis has a role in uterine relaxation during repositioning, it can aggravate postpartum hemorrhage which is undesirable in the presence of shock. As already discussed in the earlier part of the discussion, the rate of postpartum hemorrhage and shock in uterine inversion is very high. So, in practice, an early decision for general anesthesia is beneficial than giving tocolysis to a conscious woman [14]. Terbutaline, nitroglycerine and magnesium sulfate are usually used for tocolysis.

The surgical procedures used for repositioning are either abdominal or vaginal. Huntington et al. [16] described the abdominal procedure for uterine replacement of the uterus. In this procedure, after doing a laparotomy, Allis clamps are placed on the uterus at the round ligaments, and a gentle upward traction is applied. Further placement of the clamps is done on the advancing fundus. When Huntington procedure alone does not suffice, Haultain procedure is used in conjunction with it. Here, along with the Huntington procedure, a horizontal hysterotomy incision is given in the posterior portion of the lower uterine wall through the cervical ring. This incision increases the size of the opening and allows facilitation of the Huntington procedure [3, 17]. The posterior incision is repaired at the conclusion of the procedure. In the vaginal approach, the Spinelli procedure, a transverse incision is made in the anterior vaginal wall above the anterior cervical lip along with anterior uterine wall. The split uterus is repositioned by applying upward pressure on the fundus. The anterior uterine wall is closed in two layers vaginally [18].

In case 1, a laparotomy was done, intraoperatively, a second-degree inversion was diagnosed. A combined abdominal and vaginal approach was used for repositioning. Vaginally, Johnsons maneuver was used in corroboration with abdominal attempts at repositioning with allis forceps. In case 2, Johnson’s maneuver was used successfully for uterine reposition. Case 3 was also reposited manually during the surgery.

Placenta should be delivered only after repositioning of the uterus to limit postpartum hemorrhage and its consequences [4]. In our patients, placenta had already been removed in cases 1 and 2 when they presented to our hospital, and in case 3, placental removal was done after uterine repositioning.

Once the reduction is attained, uterotonic drugs are very helpful to regain the uterine tone and to prevent reinversion. In our patients, oxytocin and carboprost were used for this purpose. Use of antibiotic coverage to prevent endometritis is important.

The use of bakri balloon for uterine tamponade after repositioning the uterus have been described in literature and was used successfully in case 1 [19].

It is unknown whether the mode of delivery affects the incidence of uterine inversion. Two of our patients had a uterine inversion after a vaginal delivery while one had inversion post cesarean section. Baskett et al. reported that the rate of uterine inversions was twice as high in Caesarean sections (1 in 1860 vs. 1 in 3,737 deliveries) than in vaginal deliveries [9]. However, it was noted that these cases were less severe, were associated with immediate repositioning under direct vision and in regional anesthesia. Postpartum hemorrhage was reported in 37.5% patients, but blood transfusion was not required in any of them.

The present study is a report of three cases of varied presentations and management of acute uterine inversion. Owing to the rarity of the complication, most of the studies discuss a few cases only. A higher number of cases, however, will definitely help to validate various methods of uterine reposition described in literature.

## Conclusions

Uterine inversion is a potentially life-threatening complication which can be prevented by active and careful management of the third stage of labor and avoiding cord traction prior to signs of placental separation. In its early stages it can be difficult to diagnose and can be confused with a prolapsed fibroid or cervical polyp. This is especially important in referred patients where the placenta has already been delivered. High index of suspicion facilitates early diagnosis. Quick management by either non-surgical or surgical methods can avert maternal mortality and other associated complications like shock, blood transfusion related complications and puerperal sepsis.

## References

[1] Achanna S, Mohamed Z, Krishnan M (2006). Puerperal uterine inversion: a report of four cases. J Obstet Gynaecol Res.

[2] Wendel MP, Shnaekel KL, Magann EF (2018). Uterine inversion: a review of a life threatening obstetrical emergency. Obstet Gynecol Surv.

[3] Wendel PJ, Cox SM (1995). Emergent obstetric management of uterine inversion. Obstet Gynecol Clin N Am.

[4] Parikshit DT, Niranjan MM, Nandanwar Y (2004). Pregnancy outcome after operative correction of puerperal uterine inversion. Arch Gynecol Obstet.

[5] Eddaoudi C, Grohs MA, Filali A (2018). Uterine inversion: about a case. Pan Afr Med J.

[6] Mishra S (2018). Chronic uterine inversion following mid-trimester abortion. J Obstet Gynaecol India.

[7] Coad SL, Dahlgren LS, Hutcheon JA (2017). Risks and consequences of puerperal uterine inversion in the United States, 2004 through 2013. Am J Obstet Gynecol.

[8] Baskett TF (2002). Acute uterine inversion: a review of 40 cases. J Obstet Gynaecol Can.

[9] Brar HS, Greenspoon JS, Platt LD, Paul RH (1989). Acute puerperal uterine inversion. New approaches to management. J Reprod Med.

[10] Zohav E, Anteby EY, Grin L (2020). U-turn of uterine arteries: a novel sign pathognomonic of uterine inversion. J Ultrasound.

[11] Mihmanli V, Kilic F, Pul S, Kilinc A, Kilickaya A (2015). Magnetic resonance imaging of non-puerperal complete uterine inversion. Iran J Radiol.

[12] Ward H (1998). O’Sullivan’s hydrostatic reduction of an inverted uterus: sonar sequence recorded. Ultrasound Obstet Gynecol.

[13] Occhionero M, Restaino G, Ciufreda M, Carbone A, Sallustio G, Ferrandina G (2012). Uterine inversion in association with uterine sarcoma: a case report with MRI findings and review of the literature. Gynecol Obstet Invest.

[14] Bhalla R, Wuntakal R, Odejinmi F, Khan RU (2009). Acute inversion of the uterus. Obstet Gynaecol.

[15] Thompson W, Harper MA, Chamberlain G, Steer PJ (2002). Post partum haemorrhage and abnormalities of the 3rd stage of labour. Turnbull’s obstetrics.

[16] Huntington JL, Irving FC, Kellog FS (1928). Abdominal reposition in acute inversion of the puerperal uterus. Am J Obstet Gynecol.

[17] Das P (1940). Inversion of the uterus. Br J Obstet Gynaecol.

[18] Opaneye AA (1980). Acute uterine inversion: a report of two cases. Int J Gynaecol Obstet.

[19] Ida A, Ito K, Kubota Y, Nosaka M, Kato H, Tsuji Y (2015). Successful reduction of acute puerperal uterine inversion with the use of a bakri postpartum balloon. Case Rep Obstet Gynecol.

